# Iatrogenic empyema secondary to the malposition of a nasogastric tube

**DOI:** 10.1002/rcr2.1039

**Published:** 2022-09-11

**Authors:** Daryl Emery Chee Yeow Chan, Verena Mansour, Aaron Ting, Saurabh Gupta, Anthony Frankel

**Affiliations:** ^1^ Respiratory Department Bankstown Lidcombe Hospital Sydney New South Wales Australia

**Keywords:** empyema, fibrinolysis, nasogastric tube, pneumothorax, stroke

## Abstract

Blind NGT insertion can lead to tube misplacement into the respiratory tract. This can lead to multiple pulmonary complications including lung injury, pneumothorax, pneumonia, empyema and diaphragmatic injury. We present the case of an 80 year‐old female who required an NGT insertion for severe oropharyngeal dysphagia from an acute stroke. Her admission was complicated by multiple pneumothoraces from repeated insertions, as well as the unusual complication of a recurrent nasogastric feeding‐related empyema requiring multiple intercostal chest drain insertions for drainage. This case highlights the importance of careful NGT insertion and placement with imaging confirmation before use.

## Graphical abstract

Nasogastric tube insertions can lead to pulmonary complications if misplaced into the tracheobronchial tree. We describe a patient who has had multiple NG tubes misplaced into the left main bronchus causing pneumothoraces and an NG feeding‐related empyema.

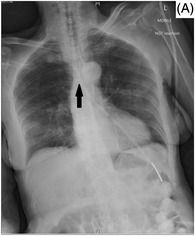

## INTRODUCTION

Misplacement of a nasogastric tube (NGT) into the respiratory tract is a relatively common complication of blind NGT insertion. This can potentially lead to severe, life‐threatening pulmonary complications. We present the case of a patient who had multiple NGT misplacements into the lung which caused recurrent pneumothoraces and NGT feeding‐related empyemas. This highlights the importance of identifying correct positioning of the NGT, careful review of imaging prior to using the NGT as well as the consideration for alternative approaches to both NGT insertions and feeding in challenging cases.

## CASE REPORT

An 80‐year‐old female was admitted to hospital for a left frontoparietal haemorrhagic stroke. She presented with right‐sided hemiplegia, facial droop and dysarthria. She had a background of severe emphysema with a 50 pack‐year smoking history. Due to severe oropharyngeal dysphagia and the consequent aspiration risk, she required an NGT to be inserted for feeding and medication administration. She was unable to obey commands due to delirium. This resulted in multiple insertion attempts, self‐removals of NGTs after successful insertion, and pulmonary misplacements of NGTs.

**FIGURE 1:**
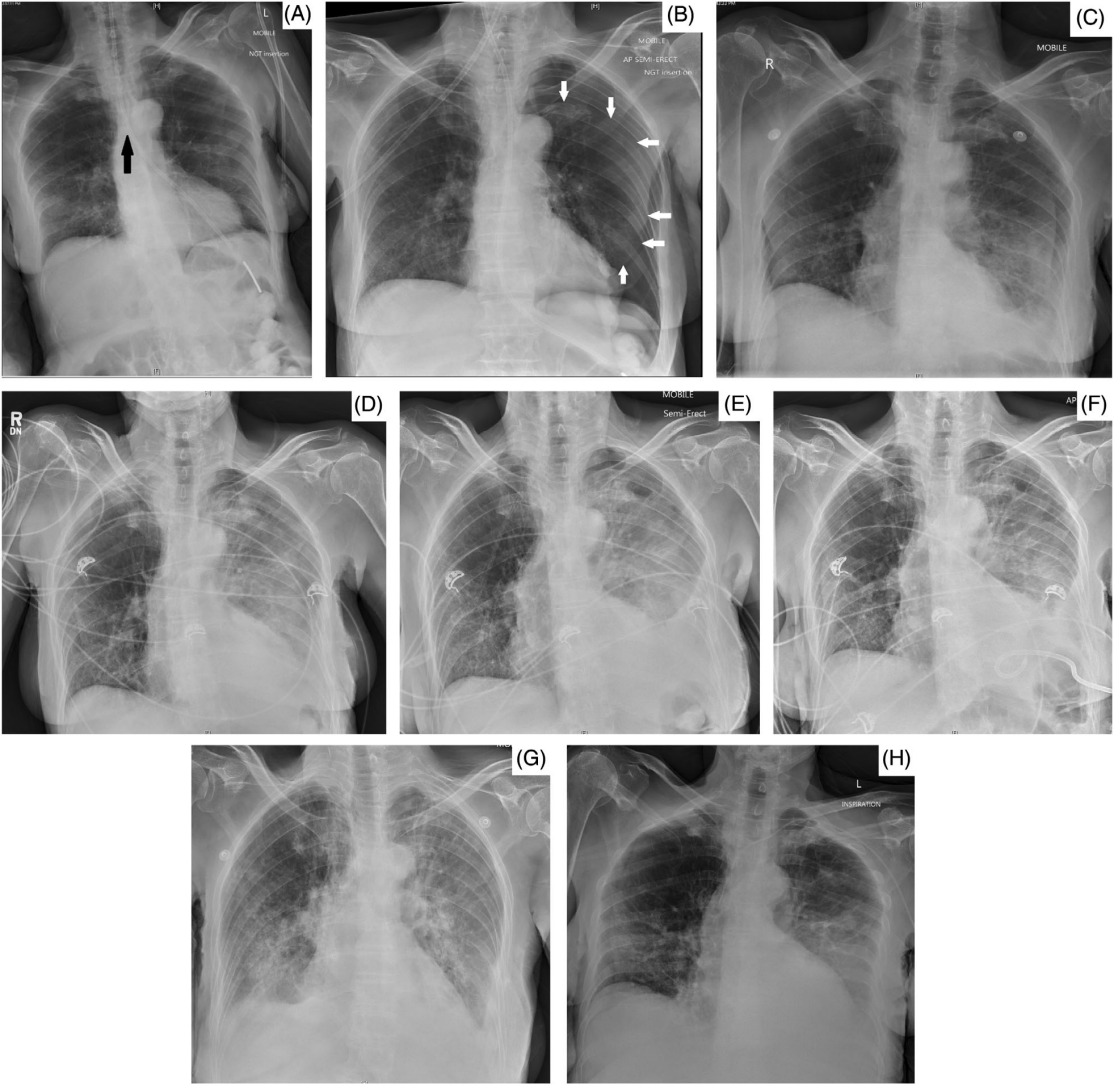
Chest X‐rays showing 1st NGT insertion through left main bronchus—black arrow denotes the sudden deviation of the NGT to the left indicating pulmonary misplacement into the left main bronchus (A), pneumothorax following 1st NGT insertion—white arrows delineating the pneumothorax after removal of the NGT (B) and serial chest X‐rays showing progression of empyema following instillation of NG feeds after the 2nd complicated NGT insertion (C–H). (D) Day 1 after removal of NGT following pleural NG feed instillation and CT identification of NGT in the lung base abutting the hemidiaphragm. (E) Day 2. (F) Day 3. (G) Day 3, following intercostal chest drain insertion. (H) Day 8, following intercostal chest drain removal after drainage has been maximized. (I) Day 15, showing improvement of empyema. There are persistent bilateral lung infiltrates from persistent aspiration secondary to oropharyngeal dysphagia

During the admission, she had two significant episodes of pulmonary misplacement of the NGT. The first episode led to transbronchial lung perforation and pneumothorax of the left lung requiring intercostal chest drain insertion (Figure 1). After several days, the pneumothorax resolved and the chest drain was successfully removed.

**FIGURE 2:**
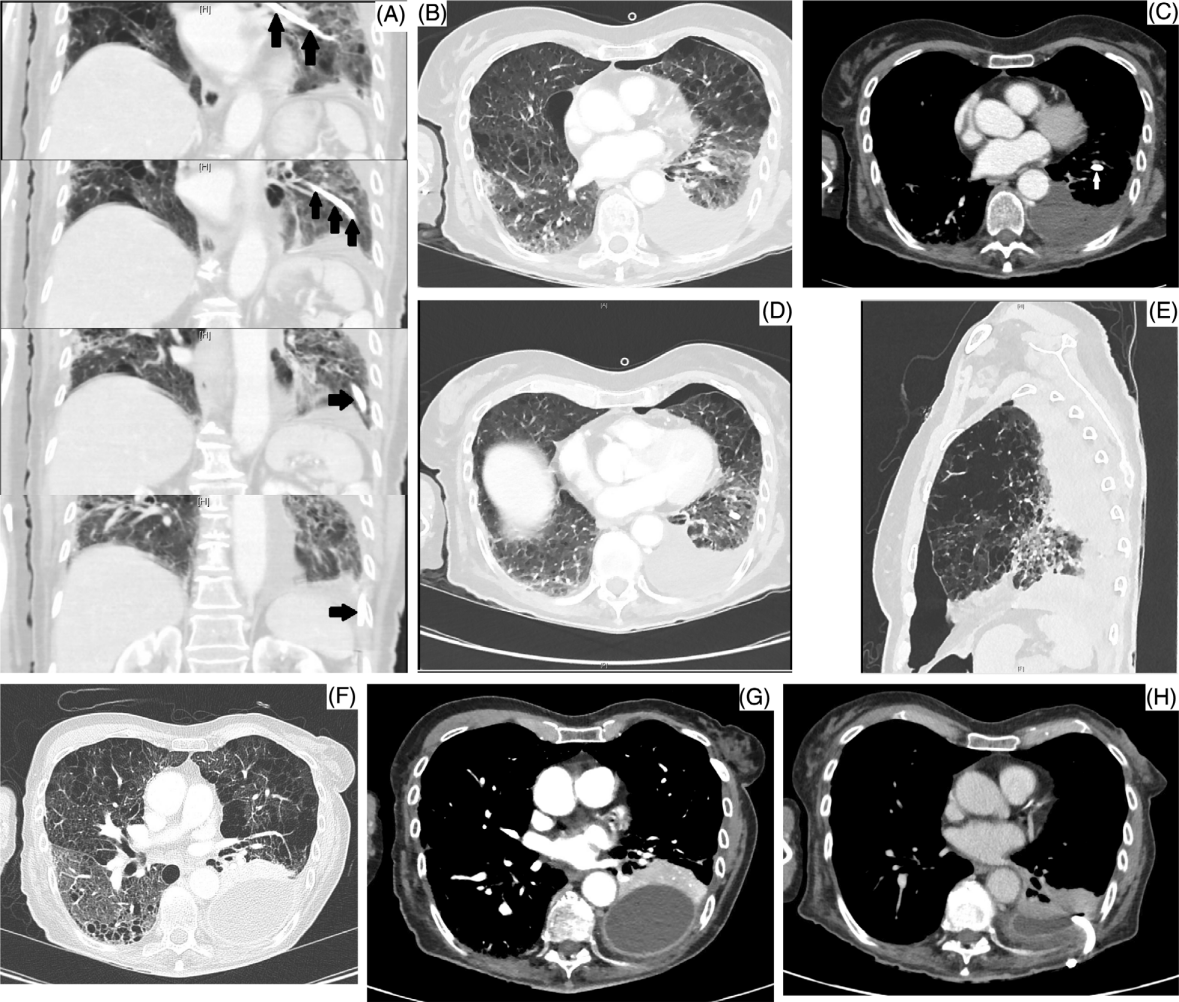
(A‐E) Initial CT Abdomen and subsequent CT Chest on Day 1 of initial empyema. (A) CT Abdomen series showing NGT inserted through left main bronchus tracking down to the base of the lung. (B, C) CT Abdomen showing significant amount of NG feeds in the pleural space at the left lung base. White arrow showing the NGT within the left lower lobe bronchus (Lung view and mediastinal view, respectively). (D, E) Subsequent CT Chest done 3 h later showing progression of pleural effusion and hydropneumothorax following NGT removal. Also noted is the significant amount of emphysema throughout both lung fields. (F, G) CT Chest showing 2nd recurrence of empyema (Lung view and mediastinal view, respectively). (H) CT Chest showing post drainage and intrapleural fibrinolysis improvement of 2nd empyema

Two weeks later, another NGT was misplaced into the left lung. The position of the NGT was misidentified on chest x‐ray as being in the stomach (by identifying the NGT tip to be below the diaphragm) and nasogastric feeds were commenced (Figure 1). After the patient received about 100 mls of feeds, she became hypoxic and developed severe abdominal pain. A CT abdomen and pelvis (with visualization of the lung bases) was performed, identifying the NGT in the left lower lobe of the lung with evidence of a small pleural effusion (due to the NG feeds in the pleural space) and pneumothorax. The NGT was removed and the patient was admitted to the intensive care unit. A follow‐up CT chest was performed on the same day which confirmed a left hydropneumothorax (Figure 2). After 2 days of monitoring, the pleural effusion increased in size and blood tests showed rising inflammatory markers (Figure 1). Her serum white cell count was 26.9 × 10^9^/L with predominantly neutrophilia (23.8 × 10^9^/L) and her C‐reactive protein was elevated at 443.8 mg/L. Another intercostal chest drain was inserted and the pleural fluid drained was typical of an empyema. The fluid pH was <6.3, lactate dehydrogenase level was 2550 U/L (serum LDH was 344 U/L and upper limit of normal for serum LDH was 250 U/L), protein level was 26 g/L (serum protein level was 53 g/L) and glucose level was <0.1 mmol/L. She was commenced empirically on a 2‐week course of intravenous moxifloxacin (due to a penicillin allergy). Subsequently, the pleural fluid culture grew methicillin‐resistant staphylococcus aureus (MRSA) and enterococcus faecalis. Both organisms were sensitive to vancomycin hence intravenous vancomycin was added to the regimen. During her admission, the progress of her empyema was monitored with serial chest X‐rays. A total of 1500 ml of pleural fluid was drained and the chest drain was removed after she had clinically improved and there was minimal output from the drain (Figure 1). By this stage, she was formally assessed with a barium swallow to be able to have oral intake and did not require any further NGTs.

With guidance from the Infectious Diseases team, her treatment was then stepped down for a 2‐week course of oral moxifloxacin whilst continuing intravenous vancomycin; this regimen was then stepped down to a single agent, oral linezolid, for a 2‐week course and ceased. She was monitored thereafter; the empyema had clinically and biochemically resolved.

Two months later, during her period of inpatient rehabilitation, she was reviewed for rigours and new oxygen desaturations. A CTPA was performed which confirmed a recurrence of the left‐sided empyema (Figure 2). Another intercostal drain was inserted and the pleural fluid culture grew enterococcus again. She received a course of intrapleural fibrinolysis (Alteplase and Dornase) as well as a further course of antibiotics; intravenous cefepime for 3 days with oral linezolid for 7 days empirically, which was rationalized and escalated to intravenous daptomycin for 3 weeks and then ceased. She recovered and was successfully discharged from hospital (Figure 2).

## DISCUSSION

NGT insertion is a common bedside procedure. There are multiple indications for nasogastric tube (NGT) insertion including gastrointestinal decompression, lavage, medication administration, enteral feeding and upper gastrointestinal bleeding. Occasionally, there may be technical challenges requiring insertion to be done by fluoroscopic or endoscopic guidance. NGT insertion can lead to complications including: malposition, coiling or knotting of the NGT in the gastrointestinal system, increased risk of gastro‐oesophageal reflux, oesophagitis, pulmonary aspiration due to impairment of the lower oesophageal sphincter, and pulmonary complications from misplacement of the NGT into the respiratory tract. Furthermore, specific pulmonary complications may include lung injury, pneumonia, pulmonary abscess, perforation, and pneumothorax. Pulmonary complications due to NGT insertion have been reported to occur in up to 8% of cases.^1^ In our patient, the risk of pulmonary misplacement of the NGT was increased due to her severe oropharyngeal dysphagia and delirium. The technical challenges presented by the complications of her stroke and her recurrent self‐removals resulted in numerous insertion attempts and each attempt increased the risk of developing pulmonary complications from misplacement. In the case of patients with severe oropharyngeal dysphagia and impaired airway protection (impaired gag, cough and swallow reflexes), early consideration of fluoroscopic or laryngoscopic techniques is advised to avoid pulmonary misplacement.

Due to the potentially life‐threatening complications of an NGT insertion, radiological confirmation of NGT position following insertion is important, especially prior to administering enteral feeds or medications.^2^ Administering nutrient‐rich feeds through a misplaced tube into the respiratory tract with concurrent introduction of naso‐oropharyngeal flora can lead to pulmonary infections such as a pneumonia, empyema, or pulmonary abscess. NGT feed‐related empyemas are rare and only described in scattered case reports.^3^, ^4^ They can occur either by direct intrapleural infusion of feeds or secondary to oesophageal perforation and mediastinitis.^5^ Regardless of the way NGT feeding causes pulmonary complications, accurate identification of the location of the NGT is paramount to avoiding them. There have been many ways of assessing placement of NGTs including clinical assessment (for coughing or choking), auscultation for gas insufflation through the NGT, assessment of NGT aspirate (visual and biochemical testing of pH, trypsin, pepsin, and bilirubin), spring gauge pressure manometry, capnography, or radiography.^6^ The most common method to identify NGT location in our centre (and many other centres in Australia) is radiography. This case highlights the importance of accurate identification of not only identifying the NGT tip to be below the diaphragm, but also following the path of the NGT as it traverses the thoracic space into the stomach. Imaging in two separate planes (i.e., anteroposterior and lateral views) may have better delineated the path of the NGT and identified it to be still in the thoracic space posterior to the diaphragm. If there is still doubt about the position of the NGT, further assessment can potentially be made with CT imaging prior to use. Auscultation for gas insufflation is another commonly used method for assessment of NGT placement. However, as pointed out by Chau et al, this method is not specific and gas insufflation into the lungs may still be heard over the epigastrium which will incorrectly identify NGT position to be in the stomach.^6^ Other methods of identification of NGT position (NGT aspirate assessment, manometry, or capnography) are also subject to availability in individual centres and expertise in interpretation.

When feeds are inadvertently administered into the pleural space, drainage by tube thoracostomy is usually required. Given that this occurs infrequently and is usually detected on clinical deterioration, there have not been any recommendations as to the timing of tube thoracostomy after detection of feeds in the pleural space. Given the progressive worsening of our patient's pleural effusion and biochemical markers with the 2 days of monitoring, we would recommend an approach of early drainage when safe to do so as opposed to monitoring for clinical deterioration.

In conclusion, it is important to be aware of the potential complications of NGT insertion, especially pulmonary complications. In technically challenging situations, it is advisable that blind NGT insertions are avoided, and radiologically or endoscopically guided insertion techniques employed to avoid pulmonary malposition of the tube. Correct identification of the position of the NGT on chest imaging is of paramount importance prior to commencing use of the tube. If foreign material is accidentally administered into the pleural space, early treatment and drainage is recommended.

## CONFLICT OF INTEREST

None declared.

## ETHICS STATEMENT

The authors declare that appropriate written informed consent was obtained for the publication of this manuscript and accompanying images.

## Data Availability

Data sharing not applicable to this article as no datasets were generated or analysed during the current study
